# A steerable introducer‐assisted wire‐loop snare technique: A bailout for unsuccessful lead extraction by a Needle's Eye Snare

**DOI:** 10.1002/joa3.12766

**Published:** 2022-08-09

**Authors:** Tsuyoshi Isawa, Taku Honda, Kazuhiro Yamaya, Shigeru Toyoda

**Affiliations:** ^1^ Department of Cardiology Sendai Kousei Hospital Sendai Japan; ^2^ Department of Cardiovascular Surgery Sendai Kousei Hospital Sendai Japan; ^3^ Department of Cardiovascular Medicine Dokkyo Medical University Mibu Japan

**Keywords:** Agilis NxT steerable introducer, lead extraction, Needle's Eye Snare, transfemoral approach, wire‐loop snare

## Abstract

The Needle's Eye Snare (Cook Medical) is an effective tool for extracting leads via a femoral vein. However, it sometimes fails to grasp the lead. We describe an alternative method of successfully grasping a lead by creating a wire‐loop around the lead with the help of a steerable introducer.

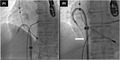

The Needle's Eye Snare (Cook Medical) is an effective tool for extracting leads via the femoral vein. Its advantage is the ability to grasp a lead without a free end, which enables a combined superior and femoral approach.^1^ However, it sometimes fails to grasp the lead. We describe an alternative method of successfully grasping a lead by creating a wire loop around the lead with the help of a steerable introducer.

A 63‐year‐old man with a complete atrioventricular block was referred to our hospital for lead extraction and upgrade to a device for cardiac resynchronization therapy. Thirteen years earlier, he had undergone an implantation of a ventricular pacing lead (Brilliant S+ VDD, IMW 15Q 58 cm; Vitatron). Transvenous lead extraction was attempted in a hybrid operating room by dedicated cardiologists and cardiac surgeons while the patient was under general anesthesia. First, a superior approach via the implant vein was attempted; however, it was unsuccessful owing to the difficulty in coaxially aligning the powered sheath with the targeted leads. The insertion of the Needle's Eye Snare through the femoral vein was subsequently attempted, but this was also unsuccessful in catching the lead because the snare was not a steerable device. Consequently, the snare could not be coaxially aligned with the lead (Video S1). Then, we used an alternative strategy; an Agilis NxT steerable introducer (Abbott) was wrapped around the lead in the right atrium (RA), and the 0.035‐inch guidewire was advanced through the steerable introducer into an EN Snare Endovascular Snare System (Merit Medical Systems, Inc.) in the inferior vena cava (IVC; Figure 1; Video S2). Furthermore, the distal end of the guidewire was snared and withdrawn into the 7‐Fr ipsilateral femoral sheath to form a wire loop (Figure 2; Video S3). Downward traction of the lead with this wire‐loop helped the powered sheath advance over the lead via the superior approach. Consequently, the result was successful lead extraction via the superior approach (Figure 3; Video S4).

**FIGURE 1:**
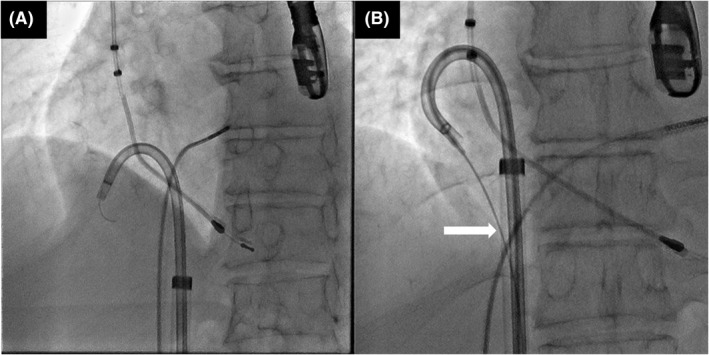
Use of the Agilis NxT steerable introducer. (A) This introducer was wrapped around the lead in the right atrium. (B) The 0.035‐inch guidewire was advanced through the introducer into the inferior vena cava (white arrow).

**FIGURE 2:**
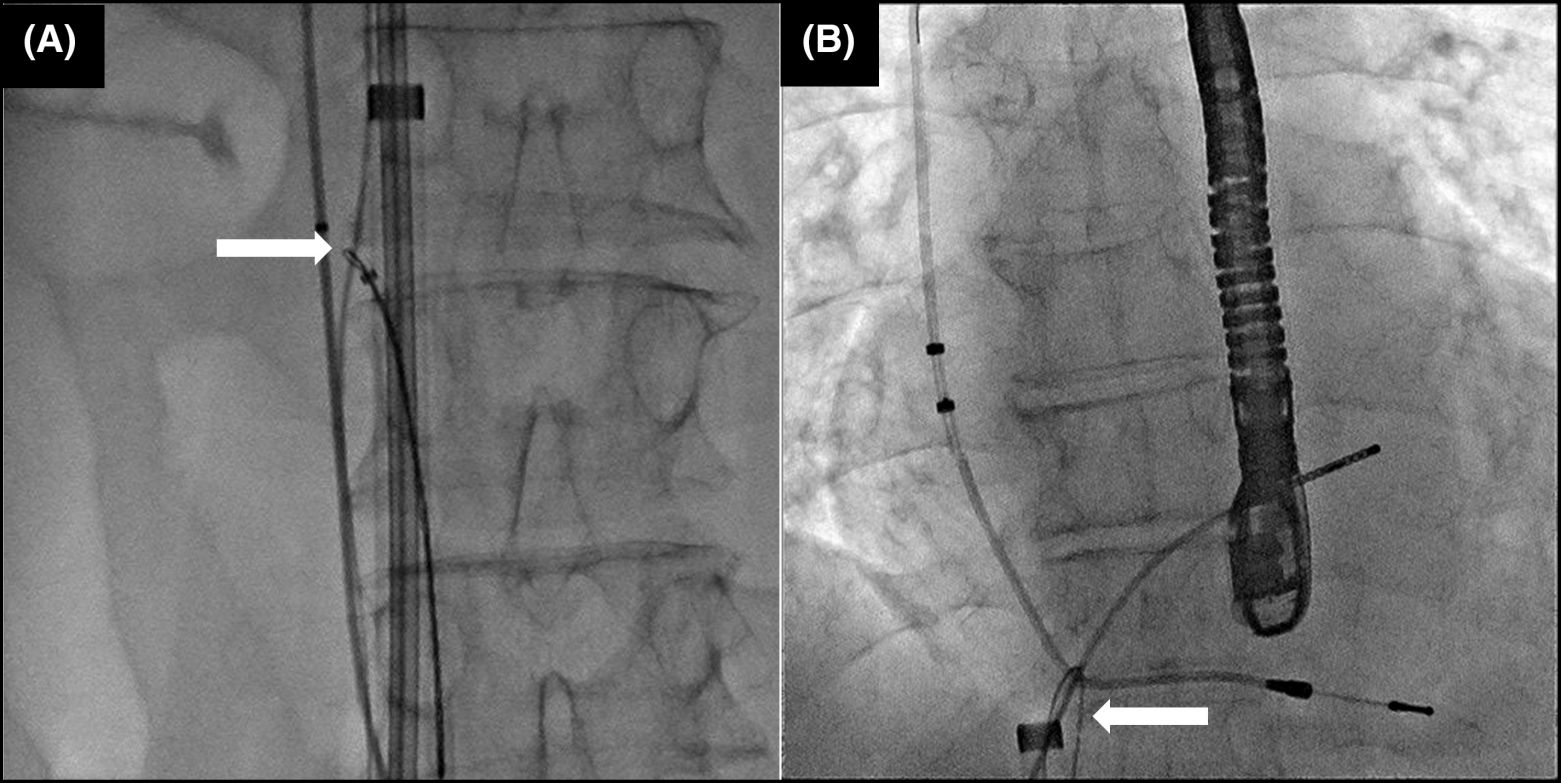
Snaring the lead. (A) The distal end of the guidewire was snared (white arrow) and withdrawn into the 7‐Fr ipsilateral femoral sheath to form a wire loop. (B) The lead was pulled caudally by the wire‐loop (white arrow).

**FIGURE 3:**
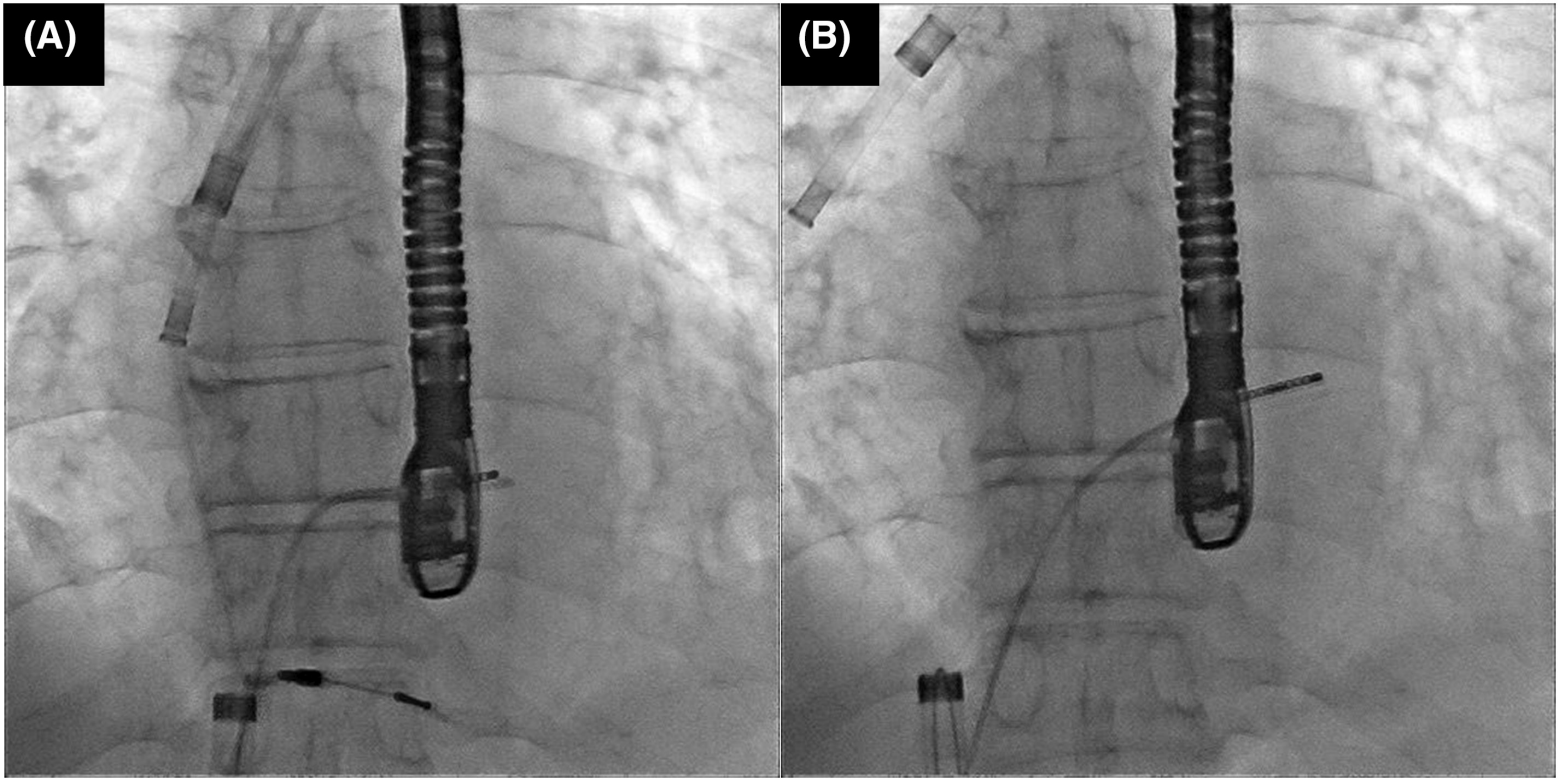
A combined superior and femoral approach. (A) The powered sheath was advanced to the superior vena cava over the lead with the aid of the wire loop via the femoral route. (B) Successful lead extraction through a superior approach.

We used an Agilis NxT steerable introducer‐assisted wire‐loop snare technique to extract a lead when a Needle's Eye Snare was unsuccessful in grasping the lead. One of the strengths of this method is the ability to encircle the lead inside the RA. Moreover, snaring the guidewire would be easier in the IVC than in the RA because an EN Snare system in the IVC would be more easily aligned coaxially with the guidewire for snaring. An alternative approach would be to catch the tip of the ablation catheter with a snare after the ablation catheter encircles the lead in the RA. Notably, this approach is similar to the technique described here, except that the location where the guidewire/ablation catheter is caught with the snare is the IVC in our approach and the RA in the alternative approach. Therefore, our approach was considered easier for snaring.

A combined superior and femoral approach has several advantages.^1^ First, it creates a strong rail to facilitate powered sheath advancement. Second, it lowers the risk of superior vena cava (SVC) laceration compared to a superior approach alone by creating a greater separation between the lead and the SVC wall. Third, this approach helps to gain cardiac chamber access by guiding a powered sheath through a venous occlusion in cases of occluded superior veins. Conversely, the disadvantage is the risk of a femoral hematoma. We believe that to extract a lead through a combined superior and femoral approach, it is critical not to unnecessarily persist with the Needle's Eye Snare since an excessive number of attempts could cause atrial injury.^2^ The Needle's Eye Snare is not useful in catching the lead when the snare is not coaxially aligned with the targeted lead or when no interstice is between a lead and SVC wall. In the latter situation, the “spaghetti twisting” technique would become difficult in catching a lead.^3^ Therefore, in these situations, an alternative to the use of the Needle's Eye Snare is necessary, as described in the present case. Additionally, the Japanese Lead Extraction registry^4^ revealed that snare techniques, which are usually used via a femoral approach, accounted for 15.2% of all lead extraction methods. Subsequently, lead extractors occasionally encounter situations where snares are required. Therefore, they should become familiar with the technique described here as a bailout for unsuccessful lead extraction using a Needle's Eye Snare.

## FUNDING INFORMATION

No funding was received for this study.

## CONFLICTS OF INTEREST

The authors declare no conflict of interest for this article.

## APPROVAL OF THE RESEARCH PROTOCOL

The clinical description of this report was approved by the Institutional Review Board of Sendai Kousei Hospital (approval no: 4‐8; approval date: May 16, 2022).

## INFORMED CONSENT

Written informed consent was obtained from the patient to publish this report.

## REGISTRY AND REGISTRATION NUMBER

N/A.

## ANIMAL STUDIES

N/A.

## Supporting information


Video S1

Video S2

Video S3

Video S4
